# Evaluation of RNA-binding motif protein 3 expression in urothelial carcinoma of the bladder: an immunohistochemical study

**DOI:** 10.1186/s12957-015-0730-3

**Published:** 2015-11-14

**Authors:** Livia Florianova, Bin Xu, Samer Traboulsi, Hazem Elmansi, Simon Tanguay, Armen Aprikian, Wassim Kassouf, Fadi Brimo

**Affiliations:** Department of Pathology, McGill University Health Centre and McGill University, Glen Site, 1001 Decarie Boulevard, Montreal, QC H4A 3J1 Canada; Department of Urology, McGill University Health Centre and McGill University, Glen Site, 1001 Decarie Boulevard, Montreal, QC H4A 3J1 Canada

**Keywords:** RBM3, Expression, Urothelial, Carcinoma, Prognosis, Metastasis

## Abstract

**Background:**

RNA-binding motif protein 3 (RBM3), involved in cell survival, has paradoxically been linked to both oncogenesis as well as an increased survival in several cancers, including urothelial carcinoma (UCA).

**Methods:**

The putative prognostic role of RBM3 was studied using cystectomy specimens with 152 invasive UCA with 35 matched metastases, 65 carcinomas in situ (CIS), 22 high-grade papillary UCAs (PAP), and 112 benign urothelium cases.

**Results:**

The H-score (HS, staining intensity × % of positive cells) was used for RBM3 immunoexpression. CIS showed the highest HS (mean = 140) followed by benign urothelium (mean = 97). Metastases showed higher HS than primary invasive UCA (*P* ≤ 0.0001), and high HS was associated with a lower pT stage (*P* ≤ 0.0001) and a trend toward the absence of lymphovascular invasion (LVI, *P* = 0.09), but not pN stage (*P* = 0.35) and surgical margin status (*P* = 0.81). Univariate analysis (UVA) of disease recurrence only showed an association between pN stage and LVI (*P* = 0.005 and 0.03, respectively). On UVA of mortality, pT stage was strongly associated with death (*P* = 0.01) while pN stage, LVI, surgical margin status, and HS were not. Multivariate analysis confirmed the lack of HS association with recurrence (*P* = 0.08) and death (*P* = 0.32).

**Conclusions:**

Stronger RBM3 immunoexpression correlated with lower stage tumors and a diminished risk for LVI. However, RBM3 does not seem to carry a prognostic significance for clinical outcome (recurrence and mortality). The exact prognostic role of RBM3 in UCA is yet to be determined.

## Background

Single-strand RNA-binding proteins (RBPs) are involved in RNA metabolism and in the regulation of the genes’ transcription [1, 2] that are essential for cell survival under adverse growth conditions such as hypoxia [3–5] and hypothermia [3]. Among these, one of the “cold-shock” proteins [6] RNA-binding motif protein 3 (RBM3), which is expressed in many fetal and adult tissues [7], has been shown to be induced during hypothermia conditions [3, 8–10] to possibly limit the decrease of protein synthesis [4, 11] and increase cell survival.

The role of RBM3 in cancer has not yet been clarified. A number of studies have suggested that RBM3 has proto-oncogenic potential as its expression was found to be up-regulated in various human tumors [3, 4]. It has been shown to increase messenger RNA (mRNA) stability and translation and to prevent apoptosis during cell mitosis (a mitotic catastrophe) [12], and to also be involved in p53-linked DNA damage repair [13]. Furthermore, RBM3 was found to be up-regulated in poorly differentiated prostate cancers in comparison to normal prostatic glands [14], as well as in low-grade vs high-grade astrocytomas [15]. A strong association has been found between RBM3 overexpression and early biochemical prostate cancer recurrence in a large series [16]. Interestingly, in a separate study [17], metastatic prostate cancer samples showed significantly decreased mRBM3 levels. Down-regulation of cold-inducible proteins including RBM3 in prostate cancer cells by exposure to hyperthermia enhanced cancer cell response to chemotherapy [13]. However, conflicting results emerged from many clinical studies which have found high RBM3 expression to be associated with a better prognosis in various cancers including colorectal cancer [18], melanoma [19, 20], estrogen-positive breast cancers [21] as well as esophageal and gastric adenocarcinomas [22]. Recently, increased RBM3 nuclear expression was also linked to cisplatin sensitivity and to an improvement in the prognosis in epithelial ovarian cancers [23, 24], as well as to a prolonged time to disease progression in prostate cancer [25]. Finally, decreased expression of RBM3 was associated with clinically more aggressive urothelial bladder cancers [26] and treatment failure in metastatic testicular non-seminomatous germ cell tumors [27].

The present study analyzes the putative prognostic role of RBM3 in a large cohort of patients with urothelial carcinoma (UCA) of the bladder by analyzing RBM3 immunohistochemical expression in various benign urothelial tissues, as well as in invasive and metastatic urothelial lesions.

## Methods

### Case characteristics

Eight tissue microarrays (TMAs) were constructed by using cystectomy tissues obtained from the surgical pathology archives of the McGill University Health Centre (Montreal, Quebec, Canada). Institutional ethical guidelines regarding experimentation involving human tissues were followed. Collectively, the samples represented 112 benign (101 normal urothelium cases and 11 normal prostate cases) and 274 malignant urothelial lesions (65 carcinoma in situ (CIS) cases, 22 high-grade papillary urothelial lesions, 152 invasive, and 35 metastatic lesions) obtained from cystectomy specimens collected between 2000 and 2012. Each case was represented by two separate 1-mm representative cores. Using the American Joint Committee on Cancer (AJCC), 51 primary tumors (35 %) were classified as pT1 or pT2 and 95 primary tumors (65 %) were classified as pT3 or pT4. Thirty-five cases were primary invasive and matched metastatic tumors from the same patient. Among invasive cases, 96 (64 %) specimens were classified as pN0 and 51 (34 %) as pN1/N2/N3; 5 cases were designated as pNx in the original pathology report. Sixty-three (41 %) cases showed lymphovascular invasion (LVI) while 90 (59 %) did not. Clinical follow-up was available in 106 patients with a mean duration of 2 years follow-up. Twenty-two percent of patients recurred while 17 % died of the disease.

### Immunohistochemistry

Immunohistochemistry was performed by using a Ventana automated system and Ventana iVIEW DAB detection kit (Ventana Medical Systems, Inc, Tucson, Arizona) at the immunohistochemistry laboratory of McGill University Health Centre. Mouse antihuman monoclonal RBM3 antibody AMAb90655 (Atlas Antibodies, Stockholm, Sweden) was used. Nuclear staining intensity was evaluated semi-quantitatively by two independent evaluators (LF and FB) as follows: 0 = negative, 1 = weak staining, 2 = moderate staining, and 3 = intense staining. The few discrepancies that existed between the two evaluators were discussed, and a consensus was reached. For each core, the percentages of stained tumor cells of each intensity were estimated. The H-score, defined as the sum of the product of the percentage of tumor cells showing RBM3 labeling (0–100) multiplied by the labeling intensity (0–3), was calculated. For statistical purposes, the H-score results of sample duplicates were averaged in order to obtain a single value.

### Statistical analysis

The data for continuous variable is expressed as means (SD). Student’s *t* test or Mann-Whitney test was used, as appropriate, to determine differences in continuous variables. Categorical variables are presented as percentage. Pearson’s chi-square test or Fisher’s exact test, as appropriate, was used to determine the differences in categorical variables. Both univariate and multivariate logistic regression analyses were performed to determine the association of RBM3 expression and various pathological parameters with recurrence and survival. From the univariate analysis, variables with *P* < 0.05 and those which were already established as significant were included in the multivariate logistic regression analysis. *P* values <0.05 were considered statistically significant. All analyses were performed using the SAS version 9.1.3 Service Pack 4 statistical (Windows platform).

## Results

### RBM3 in different histological stages of UCA

**Fig. 1:**
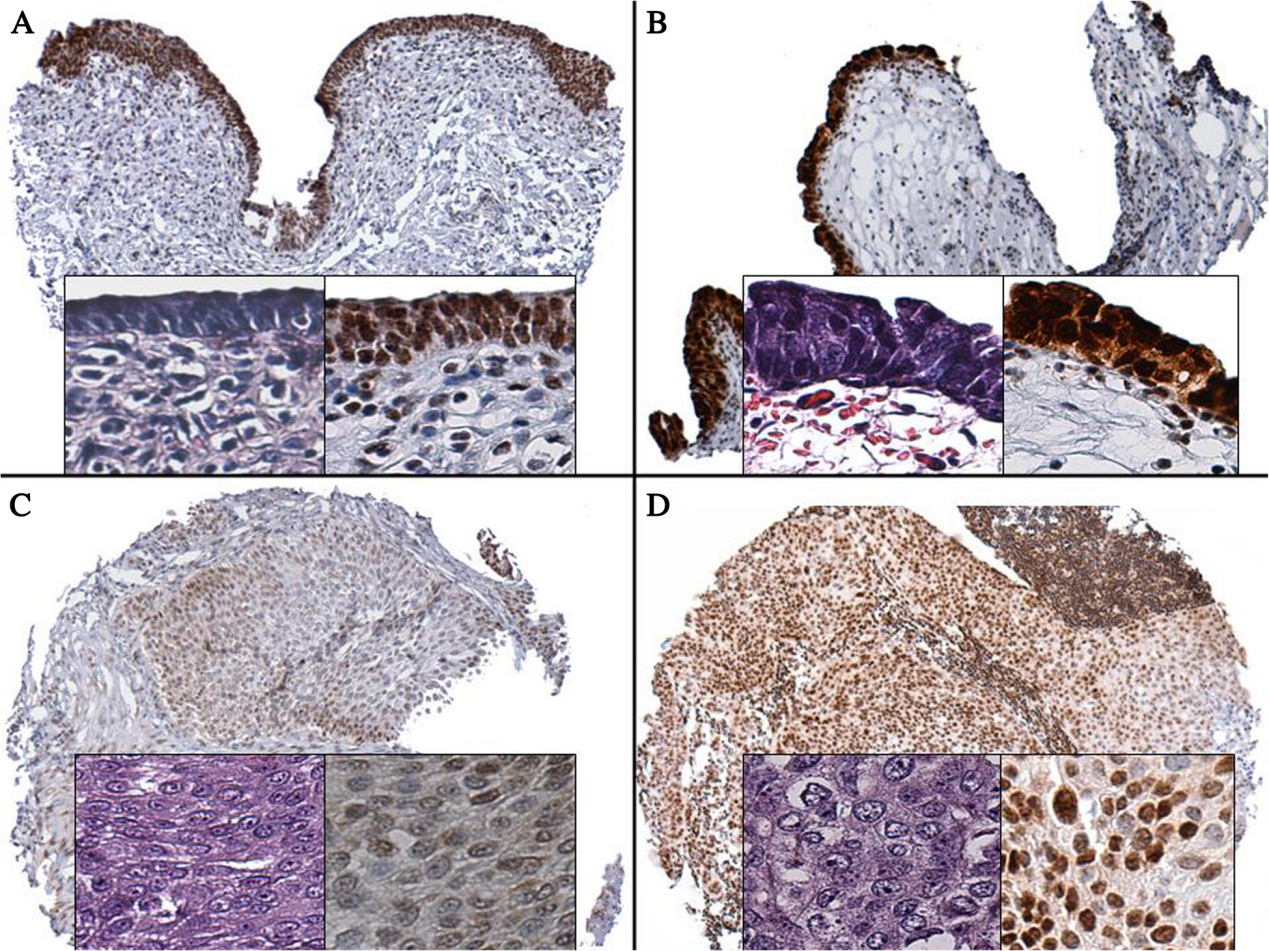
RBM3 immunoexpression and H&E morphology in benign urothelium (**a**), urothelial carcinoma in situ (**b**), primary invasive urothelial carcinoma (**c**), and matched metastatic urothelial carcinoma (**d**)

Among the non-invasive lesions, CIS showed the highest H-score (HS) (mean = 140 ± 107, *P* = 0.004), followed by benign urothelium (mean = 97 ± 69) and papillary carcinoma (PAP) (mean = 64 ± 71). The mean HS was 51 ± 59 for invasive lesions and 95 ± 87 for metastases. For matched cases, the HS was significantly higher in metastases (mean = 96 ± 87) than in invasive samples (mean = 26 ± 87) (*P* ≤ 0.0001) (Fig. 1). Detailed results are provided in Table 1.

**Table 1:** RBM3 immunoexpression in different urothelial lesions

	Number	Highest stain intensity (%)				Staining intensity (mean)	% of RBM3 labeling (mean)	H-score (mean)
		0	1	2	3			
Benign urothelium	112	8 (7 %)	21 (19 %)	24 (22 %)	58 (52 %)	2.2	61.1	97.3
Malignant urothelium	274							
CIS	65	4 (6 %)	12 (18 %)	15 (23 %)	34 (52 %)	2.2	62.5	140.7
PAP	22	5 (23 %)	5 (23 %)	4 (18 %)	8 (36 %)	1.7	37.9	64.0
Invasive	152	39 (25 %)	28 (18 %)	34 (22 %)	52 (34 %)	1.6	40.2	40.2
Primary (matched)	35	14 (40 %)	14 (40 %)	3 (9 %)	4 (11 %)	0.9	29.3	26.1
Metastasis (matched)	35	6 (17 %)	5 (14 %)	6 (17 %)	18 (51 %)	2.0	50.9	95.6

*CIS* carcinoma in situ, *PAP* papillary carcinoma

### RBM3 in relation to pathological stages of UCA

Of the cystectomy pathological parameters, RBM3 overexpression was significantly associated with a lower pT stage (*P* ≤ 0.0001) even though a significant proportion of these samples did not show a predominantly high-intensity RBM3 staining (*P* = 0.003). No association was found between RBM3 expression and pN stage (*P* = 0.35) nor with the surgical margin status (*P* = 0.81). RBM3 overexpression showed a trend toward the absence of lymphovascular invasion (*P* = 0.09).

### Recurrence analysis

On univariate analysis, pN stage and lymphovascular invasion were strongly associated with recurrence (*P* = 0.005 and 0.03, respectively). In comparison, pT stage showed a trend toward significant association (*P* = 0.1) while the HS and surgical margins status showed no association (*P* = 0.49 and 0.97, respectively). Multivariate analysis (MVA) confirmed the lack of association between the HS and recurrence (*P* = 0.08) but failed to confirm the association of recurrence to pN stage (*P* = 0.69) and to LVI (*P* = 0.08).

### Mortality analysis

On univariate analysis, pT stage was strongly associated with death of urologic cause (*P* = 0.01), while pN stage (*P* = 0.17), lymphovascular invasion (*P* = 0.28), surgical margin status (*P* = 0.18), and HS were not (*P* = 0.5). The lack of association between the HS and death was maintained using a MVA model (*P* = 0.32).

## Discussion

This study examines the immunohistochemical expression of RBM3 in various benign and malignant urothelial tissues. The RBM3 HS was highest in CIS lesions, and it was also increased in metastases when compared to invasive lesions. A high HS was also associated with a lower pT stage and trended toward showing the absence of LVI. RBM3 expression was not associated with pN stage, nor with recurrence or death on both univariate and multivariate analyses.

The literature concerning the exact role of RBM3 in carcinogenesis has so far been conflicting. RBM3 is a hypothermia-induced protein [3, 8–10] that has been found to assist in mRNA stability [12] and to increase translation efficiency in conditions of mild hypothermia [8, 12]. In turn, these events minimize DNA damage and reduce apoptotic events [12]. RBM3 mRNA and protein expression are up-regulated in hypoxia in a hypoxia-inducible factor 1 (HIF-1) independent manner [5] and increase cellular protein synthesis in conditions of cellular starvation [4]. Down-regulation of RBM3 mRNA and protein by small-interfering RNA (siRNA) reduces cell proliferation and viability in human embryonic kidney cells [4]. Thus, RBM3 appears to increase cell survival and proliferation, particularly in specific adverse growth conditions, and appears to be driving oncogenesis in various cancers. In many studies, tumor cells from various tissues including the colon, breast, pancreas, lung, ovary, and prostate displayed a higher percentage of RBM3-positive cells by immunohistochemistry as well as by RT-PCR and Western blotting in comparison to their benign counterparts [4, 12], with different studies reporting various magnitudes of expression up-regulation (from 2.4-fold [14] to 10-fold [12]). Furthermore, RBM3 was reported to be associated with tumors’ grades as poorly differentiated prostate tumors [14] and high-grade astrocytomas [15] showed higher mRNA and immunoexpression of RBM3 when compared to well-differentiated and low-grade tumors. In addition, an increased vulnerability of cells to chemotherapy was noted after down-regulation of RBM3 in prostate cancer [13]. Concurrent with this portion of the literature, our results in urothelial tissues show that the immunohistochemical HS for RBM3 was 1.4-fold higher in CIS compared to benign tissues and that in matched cases, as well as significantly higher in metastases in comparison to invasive primary samples. These results indeed seem to suggest that RBM3 is in some way involved in oncogenesis, although the fact that CIS lesions show the highest HS of all the urothelial lesions analyzed, including invasive and metastatic lesions, is somewhat paradoxical. It is possible that CIS lesions with a high RBM3 might be more aggressive and become invasive in a shorter time frame for example; however, the testing of this hypothesis would require matched cases of chronologically evolutive lesions—benign, CIS, invasive urothelial carcinoma, and eventually metastasis—which could not be achieved in the current study. Previously, it has been argued that even though in vitro findings suggest RBM3 to be a proto-oncogene, those results would not contradict the fact that high expression of RBM3 in ovarian tumors is associated with a favorable patient outcome since this data would not have taken patient treatment into account [23]. Other studies that associate high RBM3 expression with a good prognosis in colon [18], breast [21], and urothelial [26] cancer purposely highlighted the in vitro data by Sureban et al. showing proto-oncogenic RBM3 properties [12]. To explain this seeming contradiction, some rely on the argument that the majority of other clinical studies on RBM3 and other proteins of the RBM family associate them with good prognosis [21, 26], while one study made efforts to validate the antibodies they used by epitope mapping [18].

The reviewed literature (Table 2) shows that the methods for Immunohistochemistry (IHC) evaluation of RBM3 expression in TMAs are variable and inconsistent among studies which could explain the conflicting results. Several studies [20, 21, 23–26] combined the nuclear RBM3-stained fraction with the intensity of the stain to obtain a semi-quantitative value. The nuclear fraction is usually defined into four categories, with the 1 and 2 % defining the uppermost value of the “0” category and the lowermost value of the “1” category, respectively. The distinction between these very narrow values can be difficult and may lead to a positive bias, whereby perhaps biologically insignificant or potential artifactual staining of rare cells can upgrade the sample from a negative to a positive classification. Similarly, the nuclear intensity was scored variably, with some authors using four categories (negative, weak, intermediate, strong) [20, 21, 25, 26] while others pooling the intermediate and strong [23, 24] or even the negative, weak, and intermediate categories together [19]. Some on the other hand evaluated staining intensity only, as long as a predominant portion of cells displayed some staining [15, 19]. Also frequent is the dichotomization of RBM3 semi-quantitative data into low and high categories for the purpose of survival analyses [20, 21, 24–26], often based on seemingly random or unspecified values. One single study used a regression tree analysis to separate the data [25]. In the only previous study analyzing RBM3 immunoexpression in UCA, the data was dichotomized for survival analyses and trichotomized for the analyses of clinicopathological parameters. The current study uses a similar method of combined nuclear fraction and intensity to quantitatively analyze RBM3 expression in TMA samples. However, the calculated values are used as such and the data therefore remains quantitative, as opposed to the quantitative dichotomized data described in the studies above. This method may therefore be more precise when correlating RBM3 expression to clinicopathological parameters and survival, and might also explain discrepancies related to mortality and recurrence in studies examining RBM3 expression in the colon and rectum [18], epithelial ovarian carcinoma [23], and the breast [21], which found RBM3 expression to be associated with longer overall survival and disease-free survival.

**Table 2:** Summary of the literature: RBM3 studies in human cancers

Author, year (ref no.)	Tumor type	Overall results (may include methods additional to IHC)	IHC quantification method	Data adjustment for analysis
Jonsson et al., 2011 [2]	Melanoma	RBM3 is down-regulated in metastatic melanoma, and high nuclear RBM3 expression in the primary tumor is an independent marker of a prolonged OS.	Qualitative: when present, RBM3 was expressed in >75 % of the cells, so only the intensity of the staining was scored (0–3).	Samples with scores 0, 1, and 2 pooled together and compared to samples with score 3.
Ehlen et al., 2010 [3]	Ovarian cancer	RBM3 expression is associated with cisplatin sensitivity in vitro and with a good prognosis.	Semi-quantitative (combined nuclear score): 4 scores for nuclear fraction (0–1 %, 2–25 %, 26–75 %, and >75 %) and 3 scores for nuclear staining intensity (negative, intermediate, and moderate-strong).	None.
Jonsson et al., 2011 [4]	Prostate cancer	High RBM3 nuclear expression in prostate cancer is associated with a prolonged time to disease progression.	Semi-quantitative (combined nuclear score): 4 scores for nuclear fraction (0–1 %, 2–25 %, 26–75 %, and >75 %) and 4 scores for nuclear staining intensity (negative, intermediate, moderate, and strong).	RBM3 nuclear score was dichotomized into weak vs strong using classification and regression tree analysis (survival analyses).
Jogi et al., 2009 [5]	Breast cancer	Increased nuclear expression of RBM3 was associated with a prolonged overall and recurrence-free survival.	Semi-quantitative (combined nuclear score): 4 scores for nuclear fraction (0–1 %, 2–25 %, 26–75 %, and >75 %) and 4 scores for nuclear staining intensity (negative, intermediate, moderate, and strong).	RBM3 nuclear score was dichotomized into <75 vs >75 % positive nuclear staining (calculated optimal cutoff).
Nodin et al., 2012 [6]	Melanoma	RBM3 expression is an independent prognostic factor for melanoma-specific survival but not disease-free survival in the multivariable model.	Semi-quantitative (combined nuclear score): 4 scores for nuclear fraction (0–1 %, 2–25 %, 26–75 %, and >75 %) and 4 scores for nuclear staining intensity (negative, intermediate, moderate, and strong).	RBM3 nuclear score was dichotomized into low vs high.
Ehlen et al., 2011 [7]	Ovarian cancer	Association between RBM3 expression and several cellular processes involved in the maintenance of DNA integrity.	Semi-quantitative (combined nuclear score): 4 scores for nuclear fraction (0–1 %, 2–25 %, 26–75 %, and >75 %) and 3 scores for nuclear staining intensity (negative, intermediate, and moderate-strong).	RBM3 nuclear score was dichotomized into low vs high.
Boman et al., 2013 [18]	Urothelial bladder cancer	Loss of RBM3 expression is associated with clinically more aggressive tumors and an independent factor of poor prognosis.	Semi-quantitative (combined nuclear score): 4 scores for nuclear fraction (0–1 %, 2–25 %, 26–75 %, and >75 %) and 4 scores for nuclear staining intensity (negative, intermediate, moderate, and strong).	RBM3 nuclear score was trichotomized into negative, intermediate, and high (survival analyses) and dichotomized into negative vs positive or negative-intermediate vs high (relative risk of progression or death from disease analyses).
Zhang et al., 2013 [20]	Astrocytoma	RBM3 overexpression may serve as an important molecular mechanism underlying astrocytic carcinogenesis and may have proliferative and/or proto-oncogenic functions.	Qualitative only: negative, weakly positive, moderately positive, and strongly positive.	None.
Grupp et al., 2014 [22]	Prostate cancer	High RBM3 expression is an independent poor prognostic marker in prostate cancer.	Qualitative: scores combining the staining intensity and the fraction of positive tumor cells (negative, weak, moderate, and strong).	None.
Jonsson et al., 2014 [23]	Esophageal and gastric adenocarcinomas	High expression of RBM3 independently predicts a reduced risk of recurrence and death.	Semi-quantitative (combined nuclear score): 4 scores for nuclear fraction (0–1 %, 2–25 %, 26–75 %, and >75 %) and 3 scores for nuclear staining intensity (negative, intermediate, and moderate-strong).	None for the comparison of RBM3 expression in various benign, malignant, and metastatic tissues. RBM3 nuclear score was dichotomized into low and high (survival analyses).
Olofsson et al., 2015 [24]	Testicular non-seminomatous germ cell tumors (NSGCT)	Low RBM3 expression is an independent predictor of treatment failure in metastatic NSGCT.	Semi-quantitative (combined nuclear score): 4 scores for nuclear fraction (0–1 %, 2–25 %, 26–75 %, and >75 %) and 3 scores for nuclear staining intensity (negative, intermediate, and moderate-strong).	RBM3 nuclear score was dichotomized into weak vs strong using classification and regression tree analysis (survival analyses).

Specifically concerning urothelial bladder cancer, the only study currently published on this topic reported loss of RBM3 expression to be associated with clinically more aggressive tumors and to be an independent factor of poor prognosis [26]. Using a large cohort of urothelial tumors, Boman et al. used the combined nuclear score to examine RBM3 IHC semi-quantitatively and reported reduced patient survival with decreasing levels of nuclear RBM3 expression. Based on their results, the authors postulated that RBM3 expression may be used as a marker of disease progression and that IHC assessment of RBM3 expression could become a valuable tool to more accurately predict aggressiveness of urothelial carcinoma. Although those results contradict the findings of the current study, several differences that should be taken into account exist between the two studies. RBM3 nuclear score in Boman et al. was trichotomized and dichotomized in various statistical analyses while we evaluated H-score as a continuous variable (Table 3). Also, and in comparison to our study which only included cystectomy specimens with at least muscle-invasive disease and only high-grade tumors, Boman et al. samples originated from bladder biopsies or transurethral resection specimens and included only non-muscle-invasive tumors of any grade (24 % low grade). Therefore, the concept of tumor progression and recurrence between the studies is set in two totally different clinical contexts. Another different element between the two studies are the pathological parameters that were correlated along with RBM3 with outcome in statistical models; while the current study included pT, pN, surgical margins status, and lymphovascular invasion, Boman et al. included only tumor grade and stage into account. Of note, the only two studies on bladder carcinoma including the current one did not use methods other than IHC to evaluate RBM3 expression. Additional studies using non-IHC techniques may be needed to further elucidate the role of RBM3 and to confirm its utility as a prognostic marker.

**Table 3:** Comparison of studied parameters between our study and the only other study on RBM3 in urothelial carcinoma

	Current study	Boman et al. [18]
Specimen type and tumor stage^a^	Cystectomies	Bladder biopsies
		Transurethral resections of bladder tumors (TURBTs)
Distribution of high-grade disease	274/274 (100 %)	261/343 (76 %)
Categorization of patient groups	Tumor stage	
	Lymph node involvement	Tumor stage
	Lymphovascular invasion	Tumor grade
	Surgical resection margin status	
RBM3 IHC scoring	H-score (continuous variable)	Dichotomized and trichotomized nuclear scores

^a^Patients undergoing cystectomy for urothelial carcinoma have a worse prognosis due to muscle-invasive (higher stage) disease compared to patients who can initially be managed medically (intravesicular bacillus Calmette-Guerin treatment) based on biopsy or TURBT results (non-muscle-invasive or lower stage disease)

## Conclusions

The current study shows that although stronger RBM3 immunoexpression correlates with metastases, RBM3 expression does not carry a prognostic significance with respect to clinical outcome. This result contrasts with the only other study published on RBM3 in urothelial carcinoma so far, but the major study designs’ differences make further larger studies necessary before reaching a definitive conclusion about the role of RBM3 in urothelial carcinoma. Importantly, the is a continuing need to assess whether the RBM3 IHC results at different stages of the disease only mark determining points in the biological course of the disease or whether the results indeed translate into true differences in the clinical outcome for urothelial carcinoma patients.
